# Hantavirus Outbreak, Germany, 2007

**DOI:** 10.3201/eid1405.071533

**Published:** 2008-05

**Authors:** Jörg Hofmann, Helga Meisel, Boris Klempa, Silvan M. Vesenbeckh, Robert Beck, Detlef Michel, Jonas Schmidt-Chanasit, Rainer G. Ulrich, Sebastian Grund, Gisela Enders, Detlev H. Kruger

**Affiliations:** * Charité Medical School, Berlin, Germany; †University of Tübingen, Tübingen, Germany; ‡ University of Ulm, Ulm, Germany; § University of Frankfurt, Frankfurt, Germany; ¶ Institute for Novel and Emerging Infectious Diseases, Riems/Greifswald, Germany; # University Essen, Essen, Germany; ** Institute of Virology, Infectology and Epidemiology, Stuttgart, Germany; 1 These authors contributed equally to this article.; 2 Current affiliation: Bernhard-Nocht-Institute for Tropical Medicine, Hamburg, Germany.

**Keywords:** hantavirus, Puumala virus, nephropthia epidemica, Germany, letter

**To the Editor:** Hantavirus disease (for review see [*1*]) has been reportable in Germany since 2001, according to the Federal Infection Protection Act. In this country, Puumala virus (PUUV) causes most clinical hantavirus cases, although Dobrava-Belgrade virus and Tula virus also circulate (*1*). From 2001 through 2006, an average of ≈220 cases were reported per year (incidence 0.267/100,000) with a maximum of 448 cases in 2005. In contrast, 1,687 cases were reported in 2007 (*2*). Whereas in 2005 the highest incidence of infection was in metropolitan areas (*3*), the current outbreak is focused in the rural areas in southern and western Germany. Clinical case-patients exhibit key characteristics of hantavirus disease (nephropathia epidemica): acute high fever; pain in the back, head, and/or abdomen; proteinuria; rise of serum creatinine; thrombocytopenia; and renal failure (*1*). The outbreak provided considerable numbers of clinical samples from the viremic phase and thus has enabled a molecular epidemiologic analysis of the circulating virus.

At the National Consultation Laboratory for Hantavirus Infections (Berlin), we received early-phase serum specimens from the outbreak regions for confirmation assays. In enzyme immunoassays and Western blot tests (*4*), 80 samples from patients during the early clinical phase were positive for PUUV-specific immunoglobulin (Ig) M antibodies. All IgM data were accompanied by simultaneous or subsequent detection of PUUV-specific IgG. The samples were screened for hantavirus RNA by reverse transcription–PCR (RT-PCR) (*5*). Of the 80 early-phase serum samples, 42 (53%) were RT-PCR positive. For a subset of 14 of the 42 samples, a 557-nt segment of the nucleocapsid (S) gene underwent nucleotide sequence analysis as described previously (*6*).

The Figure, **panel A**, shows a map of Germany with the residences of those patients from whom virus sequences were amplified (marked by letter H in front of the specimen number). In the phylogenetic analysis, despite a substantial evolutionary distance to PUUV strains from other parts of Europe, the virus sequences unambiguously grouped within the PUUV species (Figure, **panel B**). The few previously known human PUUV sequences from individual clinical case-patients in Germany, “Berkel” from Munsterland (*7*) and “Heidelberg” from a region located between Swabian Jura and Spessart Forest (*8*), as well as human-derived strains from a small 2004 outbreak in the Bavarian Forest (*6*), were included in this analysis. The results showed a clustering of the new viral sequences strictly according to residential areas of the patients, forming the following 4 clades: Swabian Jura (SJ), Spessart Forest (SF), Munsterland (ML), and Bavarian Forest (BF). Two different single sequences, Karlsruhe (from a region in northwestern Swabian Jura) and Essen (in southern Munsterland), represent 2 putative additional lineages.

**Figure:**
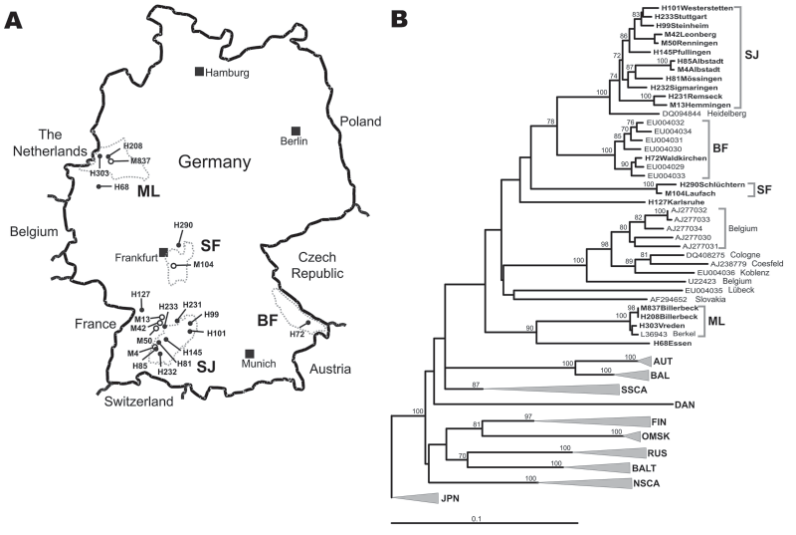
A) Map of Germany showing origins of viral sequences from the 2007 outbreak. H, sequences of human origin; M, sequences of rodent origin (*Myodes glareolus*). Dotted circles mark the outbreak regions characterized by particular virus sequence clusters; SJ, Swabian Jura; BF, Bavarian Forest; SF, Spessart Forest; ML, Munsterland. B) Neighbor-joining phylogenetic tree (TN93 evolutionary model) of European Puumala virus (PUUV) strains based on partial sequences of the S segment (557 nt, position 385–941). Bootstrap values >70%, calculated from 10,000 replicates, are shown at the tree branches. PUUV-like sequences from Japan (JPN) were used as an outgroup. Sequences taken from GenBank are indicated by their accession numbers. New sequences from this study are given in **boldface.** Accession numbers of new sequences are H101, EU266757; H233, EU266758; H99, EU266759; M42, EU085563; M50, EU085565 ; H145, EU266760; H85, EU266761; M4, EU266762; H81, EU266763; H232, EU266764; H231, EU266765; M13, EU085558; H72, EU266766; H290, EU266767; M104, EU246963; H127, EU266768; M837, EU266769; H208, EU266770; H303, EU266771; H68, EU266772. For clarity, previously characterized PUUV clades from other parts of Europe are shown in simplified form. However, the complete dataset of PUUV sequences as presented by Schilling et al. (*6*) was used to calculate the tree. Previously defined lineages are indicated by abbreviated names: AUT, Austrian; BAL, Balkan; BALT, Baltic; DAN, Danish; FIN, Finnish; NSCA, North Scandinavian; OMSK, Russian from Omsk region; RUS, Russian; SSCA, South Scandinavian. Scale bar indicates an evolutionary distance of 0.1 substitutions per position.

Most sequences in this study were obtained from Swabian Jura, the region with the highest illness rate of the outbreak (incidence 32.9/100,000). The Swabian Jura was previously identified as a hantavirus-endemic area characterized by higher seroprevalence rates in the population compared with the rest of Germany (*9*). Sequence alignments within this clade showed a nucleotide sequence diversity of up to 5.5%. Within the BF clade, the diversity is up to 4%. However, between the 4 phylogenetic clades mentioned above (SJ, SF, ML, and BF), a sequence variability of 12%–18% was found.

The natural reservoir of PUUV is the bank vole, *Myodes glareolus*; the virus is transmitted to humans by the aerosolized excreta of these rodents (*1*). Sequence comparisons showed a tight correlation between human- and rodent-derived PUUV sequences obtained from the same regional provenance (nucleotide identity >98%) and high variability of sequences originating from different geographic regions (nucleotide identity ≈85%). Neighbor-joining analyses confirmed the direct clustering of human- and rodent-derived sequences in the different phylogenetic clades (Figure, **panel B**).

In this study we focused on the analysis of a 557-nt S-segment region. For more detailed studies, analysis of the complete S and M sequences of the virus strains will be necessary. Nevertheless, our results demonstrate a high variability among the German PUUV strains but a strong clustering of viral sequences of human and rodent origin in the same geographic region. The diversity of the PUUV clusters suggests their separate evolutionary history in the different regions of Germany. In contrast, within these particular geographic areas, only slight sequence differences were found in longitudinal analysis over several years. This conclusion is supported by the novel human Waldkirchen sequence (H72), which is almost identical to the BF strains from 2004 (*6*,*10*) and the similarity of newly derived human sequences from Munsterland (H208, H303) to the Berkel strain from 1994 (*7*). The molecular characterization of the viral sequences of patient and rodent origin from the outbreak areas demonstrates that PUUV is the causative agent of the current outbreak.
